# Isolated Sacral Herpes Zoster With Urinary Symptoms: A Case Report and a Brief Review of the Literature

**DOI:** 10.1002/ccr3.73684

**Published:** 2026-10-05

**Authors:** Aida Farmani, Sarah Seyedyousefi, Mina Saber

**Affiliations:** ^1^ Department of Dermatology, Skin Diseases and Leishmaniasis Research Center, School of Medicine Isfahan University of Medical Sciences Isfahan Iran

**Keywords:** case report, herpes zoster, sacral dermatomal, urinary symptoms

## Abstract

Pure sacral dermatomal herpes zoster is a rare infectious skin disorder that can cause neurologic symptoms and a challenging diagnosis. This study highlights the potential association between urinary problems and S3 dermatomal herpes zoster and emphasizes that early recognition and management can prevent persistent neurogenic complications and significantly improve patient outcomes.

## Key Clinical Message

Isolated sacral herpes zoster is a rare condition that can potentially cause significant neurological symptoms. Early clinical recognition and management can prevent persistent neurogenic complications and improve patient outcomes.

## 1 Introduction

Herpes zoster is a clinical manifestation of reactivation of the latent varicella‐zoster virus (VZV). This virus remains in the dorsal root ganglia following primary varicella infection. Reactivation typically presents with unilateral dermatomal pain, which is followed by grouped vesicular eruptions. These eruptions most commonly involve thoracic, cervical, lumbar, or cranial dermatomes [1, 2, 3]. Recent reports note that although herpes zoster is usually limited to a single dermatome, atypical presentations, such as multidermatomal or disseminated VZV infection, particularly in older adults and immunocompromised individuals, may occur, emphasizing the importance. This emphasizes ZV reactivation [4].

Sacral involvement in herpes zoster is uncommon and accounts for approximately 4%–8% of cases [3, 5]. Because the sacral nerve roots are involved in the autonomic and somatic innervation of the bladder, bowel, and genital region, sacral herpes zoster may be complicated by urogenital and anorectal dysfunction [6, 7, 8]. Some neurological manifestations were previously reported in this regard, and include urinary retention, voiding dysfunction, constipation, erectile dysfunction, and, in rare cases, sacral radiculomyelitis or Elsberg syndrome [7, 8, 9, 10]. The diagnosis of sacral herpes zoster may be challenging, as the other early symptoms can appear before the presence of the characteristic vesicular lesions. This may mimic other common urological, gastrointestinal, or sexually transmitted conditions [6, 7, 11]. Several case reports and retrospective series have described patients presenting primarily with acute urinary retention or voiding difficulty before or concurrently with sacral dermatomal eruptions. This might lead to initial misdiagnosis and delayed antiviral therapy [6, 7, 8, 10, 11, 12].

Although urinary and anorectal symptoms are usually self‐limiting and resolve within weeks, persistent neurogenic bladder dysfunction and postherpetic neuralgia have been reported. These were particularly in cases with delayed diagnosis or severe neurological involvement [9, 10, 12]. Considering the rarity of sacral herpes zoster and its potential to cause significant urological morbidity, early diagnosis is essential and results in timely antiviral therapy and supportive management to prevent prolonged complications. Here we report a case of unilateral sacral herpes zoster presenting with urinary symptoms and provide a brief review of previously reported cases in the literature.

## 2 Case History and Examination

A 50‐year‐old woman was referred to the dermatology clinic with a 5‐day history of severe burning pain localized in the left buttock. She also reported new‐onset urinary frequency and dysuria without fever or hematuria, and denied urinary retention or urinary incontinence. She said that within 48 h of the start of the symptoms, grouped vesicles and erosions developed. There was no prior history of urinary tract infections, pelvic surgery, or known immunosuppression.

She had no history of varicella or herpes zoster vaccination and no known chronic medical conditions or recent infections, and she was not taking immunosuppressive medications.

On examination, clustered vesiculopustular lesions with erythematous bases were present in the S3 dermatomal distribution involving the left buttock and the left inner aspect of the labia major and minor. Motor strength of the lower extremities was normal; perianal sensation and anal sphincter tone were preserved. The lesions which were present at the time of examination are shown in Figure 1.

**FIGURE 1:**
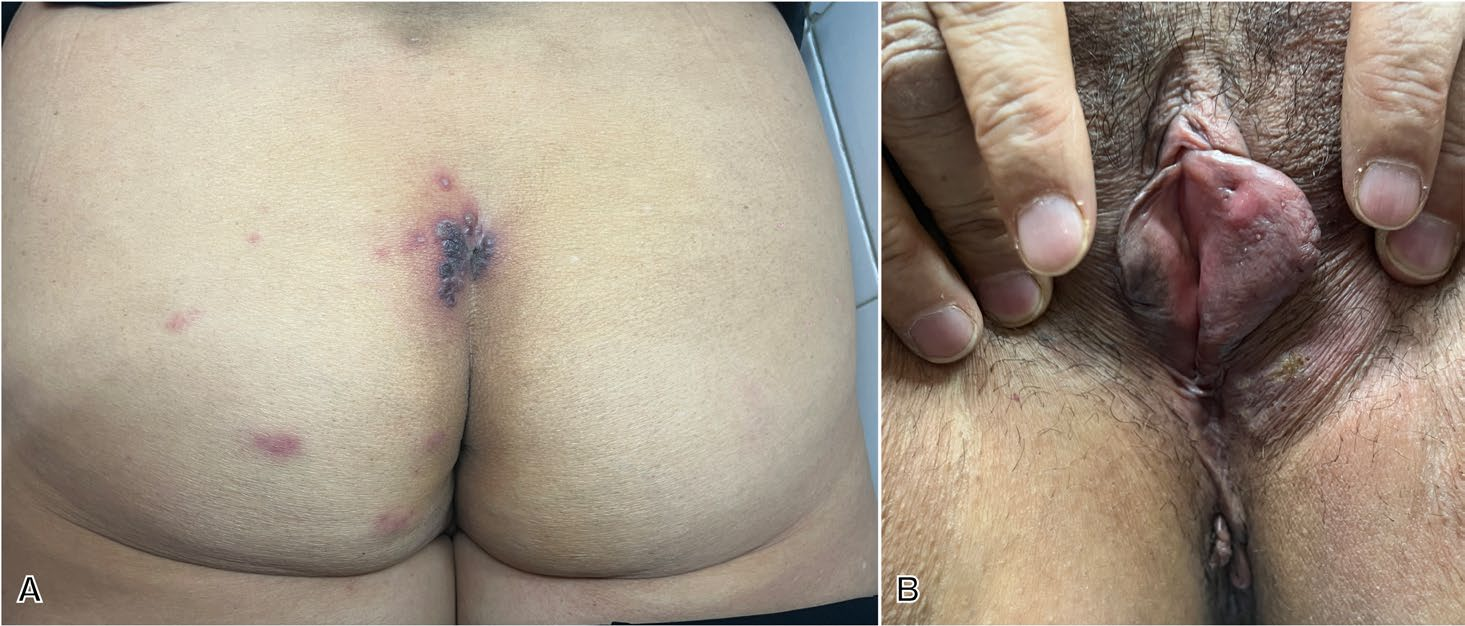
Clustered vesiculopustular lesions with erythematous bases present in the left buttock (A) and the left inner aspect of the labia major and minor (B).

## 3 Differential Diagnosis, Investigations, and Treatment

The main conditions to distinguish from S3 dermatomal herpes zoster are herpes simplex virus infection and urinary infections. Urinalysis showed no pyuria; the urine culture remained sterile. Tzanck smear showed herpetic cytopathic effects and multinucleated giant cells. Polymerase chain reaction (PCR) is the diagnostic gold standard, particularly in atypical or site‐specific presentations such as sacral herpes zoster. PCR testing was performed on a vesicular swab to confirm varicella‐zoster virus infection and to exclude herpes simplex virus infection. Given the characteristic dermatomal eruption and neuropathic pain, the Tzanck smear and the PCR, a diagnosis of sacral herpes zoster was made.

Antiviral therapy was initiated on the day of the dermatological evaluation, approximately 5 days after symptom onset.

The patient was treated with oral Valacyclovir 1000 mg three times daily for 7 days and analgesic therapy, including gabapentin titrated to symptom control. She was advised on local wound care and hygiene. No urinary catheterization was required as residual urine volumes were low and there was no severe retention. Urinary symptoms improved progressively throughout treatment and were completely resolved by two weeks. The skin lesions crusted and epithelialized by three weeks; there was no evidence of progressive neurologic deficit. At the 3‐month follow‐up, the patient reported only mild intermittent neuropathic dysesthesia with no other complications.

## 4 Discussion

Sacral herpes zoster is a rare manifestation of VZV reactivation, accounting for approximately 4%–8% of herpes zoster cases [1, 3, 5].

Its clinical importance is because of the involvement of S2–S4 nerve roots, which are connected to autonomic and somatic control of bladder, bowel, and sexual function [6, 7, 8, 13].

The pathophysiology of urinary dysfunction in sacral herpes zoster is multifactorial. The main mechanism is inflammatory neuritis, which leads to the demyelination of sacral nerve roots (S2–S4). These roots are involved in parasympathetic innervation to the detrusor muscle. This muscle is essential in the coordination of bladder emptying. Inflammatory involvement of these fibers can lead to detrusor areflexia or hypocontractility. This can result in urinary retention, incomplete bladder emptying, or dysuria [6, 12, 14, 15]. In some patients, VZV reactivation may be more severe and can involve the spinal cord or cauda equina. This can manifest as sacral radiculomyelitis or Elsberg syndrome [9, 10].

While most patients experience gradual recovery over weeks, persistent neurogenic bladder dysfunction has been reported, particularly in older individuals or those with severe neurological involvement [10, 12]. Sacral herpes zoster can be challenging to diagnose because visceral or neuropathic symptoms may precede the rash. This might lead to misdiagnosis as urinary tract infection, prostatitis, vulvovaginitis, or other pelvic pathology [6, 7, 8, 11]. In several reported cases, urinary retention or voiding difficulty was the main and first complaint, and the vesicular eruptions appeared days later [11, 12]. Clinicians should be suspicious of VZV in patients presenting with acute urinary symptoms accompanied by dermatomal pain, paresthesia, or perineal discomfort, even when rashes are still absent.

Previously reported cases of sacral herpes zoster with urogenital manifestations are summarized in Table 1.

**TABLE 1:** A summary of reported cases of sacral herpes zoster.

Study	No. of cases	Age (year) and gender	Clinical manifestations, physical examinations, paraclinics	Involved dermatome	Management	Outcome, prognosis, complications
Richmond et al. [8]	3	74 Female	Urinary retention, painful vesicular eruption involving the perineum and right buttock Urinary retention	S3	Catheter drainage	The patient voided urine spontaneously after 13 weeks
		68 Male	Severe bilateral girdle pain over the 10th thoracic dermatomes, generalized lower limb weakness and Paraesthesiae Constipation	T10	Catheter drainage	Follow‐up examination in 1 month the patient was asymptomatic
		68 Male	Urinary retention Zoster eruption involving the 2nd and 3rd lumbar dermatomes on the left side	L2/3	Catheter drainage	Catheter was removed after 10 days Normal micturition was resumed after a single intramuscular dose of carbachoasymptomatic
Erol [13]	1	41 Male	Erythematous vesicular lesions in the right testicular and perineal area, difficulty of defecation, forced urination insufficient erection Pain and light touch sensations impaired mildly through the right S2–S5 Cremaster and anal reflexes were decreased, and the bulbocavernous reflex was absent Meningeal infection by VZV Bladder areflexia	S2–S5	Parenteral acyclovir therapy was started with a dose of 15 mg/kg/day 3 times daily urinary catheter laxative	Erectile dysfunction improved on the 10th day Anorectal dysfunction on the 6th day of hospitalization. The urethral catheter was pulled out on the 15th day
Kalantzi et al. [16]	Case report 1	51 Female	Vulvar pain and burning sensation Unilateral vesicular lesions on her left labium majus, the perianal area, the medial thigh surface, and the gluteal region PCR positive for VZV	S2–S3	Brivudine 125 mg once daily for seven days local mupirocin	After one week significant improvement in symptoms
Seo et al. [14]	Case report 1	80 Male	Vesicular and crusted lesions over the right S2 and S3 dermatomes the perineal raphe Acute urinary retention Tingling sensation in the right posterior thigh Decreased anal tone Urody namic study revealed mild hypotonic detrusor (bladder areflexia)	S2–S3	Oral acyclovir for 12 days Intravenous (IV) acyclovir therapy dosing at 10 mg/kg Three times daily for 7 days, IV ceftriaxone dosing at 2 g/day for 5 days oral corticosteroid was given at 5 mg twice daily	Neuritis in S2 and S3 dermatomes resulting in acute uri nary retention with detrusor areflexia
Biglione et al. [12]	Case report 1	75 Male	Vesicular lesions on Left glans penis, ventral shaft, and scrotum to the left medial buttock Mild dysuria, difficulty voiding, decreased bowel movements, (PCR) analysis was positive for VZV	S3–S4 dermatome	5 days intravenous acyclovir 10 mg/kg every 8 h gabapentin 300 mg nightly foley catheter placed with simultaneous initiation of tamsulosin polyethylene glycol, senna, and a bisacodyl suppository	At the 3‐week follow‐up, the patient had resolution of lesions and constipation 8‐week follow‐up, a urodynamic study confirmed the diagnosis of hypotonic neurogenic bladder. The patient was started on lifelong therapy with alfuzosin for autonomic nerve damage
Nishiyama et al. [10]	Case report 1	77 Female	Genital rash voiding dysfunction, incomplete bladder emptying Abdominal distention for 10 days Immunofluorescence testing for VZVantigen from the vesicle was positive varicella zoster virus IgG titers (8 units) there was no sign of cystitis on urinalysis	S2–S3	Intravenous acyclovir 250 mg every 8 h (q8h) for 1 week	The skin rash and voiding dysfunction did not recur for 6 months Postherpetic neuralgia persisted
Grekin et al. [11]	Case report 1	27 Male	Unilateral vesicular rash the left buttock left perineal region and left posterior scrotum within the S2 to S4 dermatom difficulty with urinary voiding	S2–S4	7‐day course of oral valacyclovir (1 g, 3 times daily) tamsulosin (0.4 mg daily)	
Hur et al. [17]	Case report 1	25 Male	Skin rash on the on both sides of the anus Voiding dysfunction Constipation A Tzanck test revealed multinuclear cells	S1–S5	Acyclovir (10 mg/kg every 8 h) for seven days Dexamethasone (10 mg/day) for six days Prednisolone (100 mg oral daily).	The patient's symptoms subsided
Marquese et al. [15]	Case report 1	65 Female	Gluteal herpes zoster urinary retention constipation	S2–S4	Valacyclovir corticosteroids 0.7 mg/kg/daily	Fully recovered after eight weeks
Kogler et al. [18]	Case report 1	61 Male	Solitary erythematous macule in the gluteal area dysuria PCR (VZV) was positive	S3 dermatome	Acyclovir at 5 mg/kg Three times daily for a duration of 10 days	

The majority of reported patients had involvement of the S2–S4. Urinary retention and voiding dysfunction were the most commonly reported urological manifestations, while constipation, dysuria, incomplete bladder emptying, and sexual dysfunction were also described.

There are reports of patients with urinary or pelvic symptoms, for whom a diagnosis was established after the appearance of characteristic vesicular lesions or confirmation of VZV infection by laboratory testing.

Most patients treated with antiviral therapy (acyclovir or valacyclovir) and supportive interventions, such as urinary catheterization when required.

Some patients developed prolonged neurological complications, including neurogenic bladder, detrusor dysfunction, or postherpetic neuralgia. In particular, Seo et al. and Biglione et al. reported urodynamic evidence of bladder dysfunction, highlighting the potential for persistent autonomic impairment. Our patient presented with urinary symptoms; however, unlike in some previously reported cases, no persistent neurological complications were observed during follow‐up [8, 10, 11, 12, 13, 14, 15, 16, 17, 18].

PCR from vesicular fluid is considered the diagnostic gold standard, particularly in atypical presentations or genital/perineal involvement, as differentiation from herpes simplex virus is clinically relevant in these cases. Neuroimaging and urodynamic studies may be indicated in patients with severe or persistent neurological symptoms to evaluate for radiculomyelitis or neurogenic bladder [10, 12, 14, 16, 18].

Early initiation of antiviral therapy is critical in herpes zoster to limit viral replication. This results in a reduction of the disease severity, and a potential decrease in neurological complications [1, 6, 12]. Oral antivirals such as acyclovir or valacyclovir are appropriate in uncomplicated cases, whereas intravenous acyclovir should be considered in patients with severe neurological involvement, suspected radiculomyelitis, or significant urinary retention. Adjunctive analgesic therapy with agents such as gabapentin is commonly required for neuropathic pain control [10, 12, 14].

From a urological perspective, patients presenting with acute urinary retention require prompt bladder decompression with catheterization to prevent overdistension and secondary complications. Alpha‐blockers (e.g., tamsulosin) have been used in some cases to facilitate voiding. Bowel dysfunction may require laxatives or stool softeners. Persistent urinary dysfunction needs a urological follow‐up. Urodynamic assessments might be needed to guide long‐term management [6, 10, 11, 12, 14]. Most cases of urinary dysfunction associated with sacral herpes zoster are self‐limiting and resolve within several weeks. Delayed diagnosis, older age, and extensive neurological involvement have been associated with poorer recovery [10, 12].

Knowing this rare presentation is essential for dermatologists, urologists, and primary care clinicians, as early recognition and management of sacral herpes zoster can significantly improve patient outcomes. Considering it in patients with acute urinary symptoms and appropriate management may prevent prolonged neurological complications.

## 5 Conclusion

Sacral herpes zoster should be considered in patients with acute urinary symptoms, especially when accompanied by unilateral sacral pain or dermatomal skin lesions. Early diagnosis and antiviral treatment may help prevent neurological complications and promote recovery.

## Author Contributions

**Aida Farmani:** conceptualization, investigation, methodology, supervision, writing – original draft, writing – review and editing. **Sarah Seyedyousefi:** investigation, writing – review and editing. **Mina Saber:** investigation, supervision, writing – review and editing.

## Funding

The authors have nothing to report.

## Ethics Statement

This study was conducted in accordance with the principles outlined in the Declaration of Helsinki of the World Medical Association. It was approved by the Ethics Committee of the Isfahan University of Medical Sciences (approval code: IR.ARI.MUI.REC.1404.241).

## Consent

Written informed consent was obtained from the patient for the publication of this case report and accompanying images.

## Conflicts of Interest

The authors declare no conflicts of interest.

## Data Availability

All data supporting the findings of this case report are included in this article. Further details are available from the corresponding author upon reasonable request.
